# Soluble neprilysin and long-term clinical outcomes in patients with coronary artery disease undergoing percutaneous coronary intervention: a retrospective cohort study

**DOI:** 10.1186/s12872-020-01636-5

**Published:** 2020-08-06

**Authors:** Ik Jun Choi, Sungmin Lim, Youngdeok Hwang, Dongjae Lee, Won Jik Lee, Kwan Yong Lee, Mi-Jeong Kim, Doo Soo Jeon

**Affiliations:** 1 Division of Cardiology, Department of Internal Medicine, Incheon St. Mary’s Hospital, College of Medicine, The Catholic University of Korea, Seoul, Republic of Korea; 2 Division of Cardiology, Department of Internal Medicine, Uijeongbu St. Mary’s Hospital, College of Medicine, The Catholic University of Korea, 271, Cheonbo-ro, Uijeongbu-si, Gyeonggi-do, 11765 Seoul, Republic of Korea; 3 Paul H. Chook Department of Information Systems and Statistics, Baruch College, CUNY, New York, NY USA

**Keywords:** Neprilysin, Coronary artery disease, Percutaneous coronary intervention, Prognosis, Left ventricular ejection fraction

## Abstract

**Background:**

Neprilysin has an essential role in regulating fluid balance and vascular resistance, and neprilysin inhibitors have shown beneficial effects in patients with heart failure. However, the potential predictive value of neprilysin levels as a biomarker for cardiovascular risk remains unclear. The aim of this study was to assess the prognostic value of soluble neprilysin (sNEP) levels in patients with ischemic heart disease.

**Methods:**

Neprilysin levels were measured in 694 consecutive patients with coronary artery disease (CAD) undergoing percutaneous coronary intervention (PCI). These patients were classified into two groups according to their serum levels of neprilysin and categorized into the lower neprilysin group (*n* = 348) and the higher neprilysin group (*n* = 346). The primary clinical endpoint was all-cause mortality, and the secondary endpoint was a composite of major adverse cardiac events (MACE).

**Results:**

The median sNEP level was 76.0 pg/ml. The median sNEP levels were higher in patients with left ventricular ejection fraction (LVEF) ≥40% (77.6 pg/ml, interquartile range 46.6–141.3) than in those with LVEF < 40% (70.0 pg/ml, interquartile range 47.1–100.6; *P* = 0.032). Among all patients, each clinical outcome and MACE did not differ significantly according to the groups divided into median, tertile, or quartile of sNEP levels during a median follow-up of 28.4 months. We did not find a significant relationship between sNEP levels and clinical outcomes in multivariate Cox regression analysis. Among patients with LVEF < 40%, an increased sNEP level was associated with a higher rate of all-cause death (adjusted hazard ratio 2.630, 95% confidence interval 1.049–6.595, *P* = 0.039).

**Conclusion:**

Serum sNEP levels are not associated with long-term mortality or cardiovascular outcomes after PCI in patients with CAD. In the LVEF < 40% group, increased sNEP levels may be associated with a higher risk of all-cause death.

## Background

Neprilysin (NEP) is a zinc-dependent type II integral membrane peptidase that degrades a variety of vasoactive peptides such as atrial natriuretic peptide, brain or B-type natriuretic peptide, bradykinin, adrenomedullin, and endothelin-1 [1–3]. These vasoactive peptides play essential roles in fluid balance and vascular resistance [4–6]. Therefore, efforts have been made to inhibit neprilysin as a treatment target for heart failure (HF), and recent clinical trials have obtained remarkable results. Combined inhibition of NEP and angiotensin have been established the effective therapeutic value in patients with HF and reduced ejection fraction (HFrEF) or acute HF [7–9].

Circulating NEP levels were significantly associated with cardiovascular death or HF hospitalization in patients with HFrEF [10]. In addition, previous studies identified a positive association in acute HF [11, 12], whereas other studies did not confirm an association of soluble NEP (sNEP) and cardiovascular mortality and morbidity in patients with HF and preserved ejection fraction (HFpEF) and ST-segment elevation myocardial infarction [13, 14]. The impact of sNEP levels on clinical outcomes in patients with ischemic heart disease has not been well established. Therefore, the present study aimed to demonstrate the association between serum sNEP levels and mortality and cardiovascular events in patients with coronary artery disease (CAD) undergoing percutaneous coronary intervention (PCI).

## Methods

### Study population

Between September 2015 and Novermber 2017, 796 patients with CAD scheduled for PCI and older than 20 years were screened. Exclusion criteria were as follows: patients with cardiogenic shock, patients with end-stage renal disease and on dialysis, patients who managed conservative care without coronary intervention, and patients without sufficient blood samples. Of the 796 eligible patients, 694 had samples available for measurement of serum levels of neprilysin. All participants provided written informed consent to participate before PCI and blood sampling. The study protocol was reviewed and approved by the appropriate institutional review board.

PCI was performed according to standard techniques and left to the operators’ discretion. After the procedure, all patients were recommended to receive optimal pharmacological therapy, including dual-antiplatelets, statins, beta-blockers, or renin-angiotensin blockade, if indicated, following standard European and American guidelines [15–18]. Clinical follow-up was performed every 3 months after the index procedure.

### Laboratory measurement

Blood was drawn upon arrival at the catheterization laboratory and was collected immediately after sheath insertion and before the PCI. After the blood was centrifuged, plasma was subsequently stored at − 80 °C. Serum sNEP levels were measured by an optimized enzyme-linked immunosorbent assay (ELISA) using a High-Sensitivity Soluble Neprilysin (Human) ELISA Kit (Aviscera Bioscience, INC., Santa Clara, CA, USA). The measurement of neprilysin levels was performed in the Clinical Research Laboratory, Incheon St. Mary’s Hospital, The Catholic University of Korea.

### Study endpoints and definitions

The primary endpoint was all-cause death. The secondary endpoint was major adverse cardiovascular events (MACE), including cardiovascular death, nonfatal myocardial infarction, nonfatal stroke, any revascularization, and hospitalization for HF. Patient follow-up data, including censored survival data, were collected through March 31, 2019, via hospital chart review, telephone interviews with patients by trained reviewers who were blinded to the study results, and reviews of the database of the National Health Insurance Corporation, Korea, using a unique personal identification number.

Myocardial infarction was defined as an elevation of a cardiac enzyme level, especially high-sensitivity troponin T, above the upper limit with ischemic symptoms or electrocardiographic findings indicative of ischemia that was not related to the PCI. Stroke was defined as any nonconvulsive focal or global neurological deficit of abrupt onset lasting more than 24 h caused by ischemia or hemorrhage within the brain. Revascularization was defined as any repeat PCI which was unexpected and clinically indicated revascularization. Planed and staged interventions were not considered as repeat revascularization. All events were adjudicated by two interventional cardiologists.

### Statistical analysis

Continuous variables are expressed as the mean ± standard deviation or median (interquartile range (IQR)) and were analyzed by independent sample *t*-test or the Mann-Whitney *U*-test according to the distribution. Categorical variables are presented as percentages or rates and were analyzed by the chi-square test or Fisher’s exact test. A comparison of clinical outcomes between groups was performed with the long-rank test. Receiver operating characteristic (ROC) curve analyses were performed to identify the optimal cutoff value of neprilysin associated with clinical outcomes. Kaplan-Meier curves were used to analyze the overall survival rate and adverse events of patients. Cox proportional hazard models were applied to analyze the hazard ratio (HR) and 95% confidence interval (CI) for clinical outcomes. We examined the association between sNEP levels on a continuous scale and all-cause mortality or adverse cardiovascular events during the follow-up period using the Cox proportional hazard model. All analyses were 2-tailed, and *P* < 0.05 was considered statistically significant. All statistical analyses were performed using SPSS 20.0 statistical software (SPSS Inc., Chicago, IL, USA) and R version 3.6.1 (R Foundation for Statistical Computing, Vienna, Austria).

## Results

### Study population and characteristics

The mean age of all available 694 patients was 65.9 ± 11.9 years old, and 67.0% of the patients were men. Among them, 48.0% were diagnosed with myocardial infarction. The mean left ventricular ejection fraction (LVEF) was 55.0 ± 11.7%, and LVEF < 40% was observed in 86 (12.4%) patients. The median serum neprilysin level was 76.0 pg/ml (IQR 46.8 to 133.9). Table 1 presents the baseline characteristics according to the groups divided by the median neprilysin level. The age of the neprilysin ≤76.0 pg/ml group was significantly younger than that of the neprilysin > 76.0 pg/ml group. The prevalence of a family history of CAD was higher in the high neprilysin level group. LVEF was not different between groups. Laboratory and procedural data were also similar between the two groups.

**Table 1:** Baseline clinical and angiographic characteristics

Variables	Neprilysin ≤76.0 pg/ml (*n* = 348)	Neprilysin > 76.0 pg/ml (*n* = 346)	*P* value
Age (years)	66.8 ± 11.2	64.9 ± 12.4	0.027
Male	231 (66.4%)	234 (67.6%)	0.394
Body mass index (Kg/m^2^)	24.5 ± 4.1	24.5 ± 4.1	0.983
Hypertension	238 (68.4%)	249 (72.0%)	0.172
Diabetes mellitus	138 (39.7%)	124 (35.8%)	0.169
Dyslipidemia	123 (35.9%)	120 (35.4%)	0.482
Smoking	90 (25.9%)	110 (31.8%)	0.050
Family history of coronary artery disease	20 (5.7%)	33 (9.5%)	0.041
Chronic kidney disease	16 (4.6%)	17 (4.9%)	0.493
Prior stroke	37 (10.6%)	37 (10.7%)	0.538
Prior myocardial infarction	32 (9.2%)	23 (6.6%)	0.135
Prior percutaneous coronary intervention	46 (13.2%)	43 (12.4%)	0.422
Clinical presentation			0.982
Stable angina	19 (5.5%)	22 (6.4%)	
Unstable angina	153 (44.0%)	155 (44.8%)	
NSTEMI	110 (31.6%)	107 (30.9%)	
STEMI	60 (17.2%)	56 (16.2%)	
Silent myocardial ischemia	6 (1.7%)	6 (1.7%)	
Left ventricular ejection fraction (%)	54.5 ± 12.1	55.6 ± 11.3	0.222
Total cholesterol (mg/dl)	165.6 ± 46.3	164.2 ± 43.2	0.676
Triglyceride (mg/dl)	141.0 ± 86.0	154.1 ± 133.4	0.137
HDL cholesterol (mg/dl)	41.6 ± 10.5	41.3 ± 9.9	0.682
LDL cholesterol (mg/dl)	101.0 ± 34.1	99.0 ± 31.8	0.415
High-sensitivity C-reactive protein (mg/l)	8.4 ± 23.9	10.0 ± 28.0	0.406
eGFR (ml/min/1.73m^2^)	69.6 ± 26.4	73.8 ± 30.8	0.056
NT-proBNP (pg/ml)	1384.0 ± 4585.9	1919.1 ± 6243.0	0.199
Culprit lesion			0.628
Left anterior descending	171 (51.5%)	165 (49.8%)	
Left circumflex	62 (18.7%)	54 (16.3%)	
Right coronary artery	83 (25.0%)	95 (28.7%)	
Left main	15 (4.5%)	17 (5.1%)	
Extent of coronary artery disease			0.256
1-vessel disease	164 (49.4%)	146 (44.1%)	
2-vessel disease	100 (30.1%)	119 (36.0%)	
3-vessel disease	68 (20.5%)	66 (19.9%)	
Multivessel disease	103 (29.6%)	112 (32.4%)	0.240
Number of total stents	1.6 ± 1.0	1.7 ± 1.2	0.139
Mean diameter of stents	3.10 ± 0.43	3.11 ± 0.41	0.677
Total length of stents	42.2 ± 28.8	46.8 ± 35.4	0.065

*eGFR* indicates estimated glomerular filtration rate, *HDL* high-density lipoprotein, *LDL* low-density lipoprotein, *NSTEMI* non-ST-segment elevation myocardial infarction, *NT-proBNP* N-terminal pro-B-type natriuretic peptide, *STEMI* ST-segment elevation myocardial infarction

**Fig. 1:**
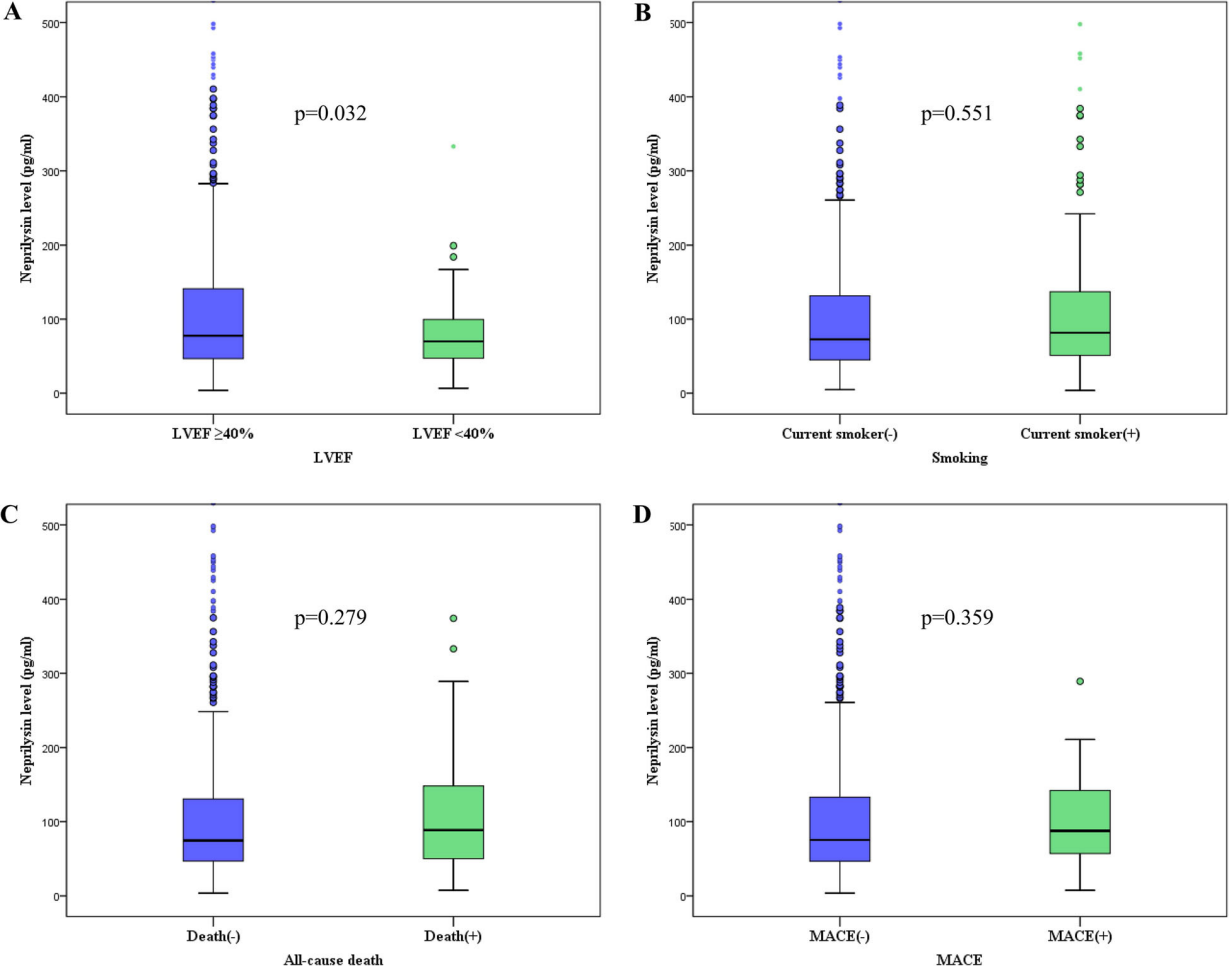
Comparison of soluble neprilysin levels. **a** Left ventricular ejection fraction (**b**) smoking (**c**) all-cause death (**d**) major adverse cardiovascular events

The median sNEP levels were higher in patients with LVEF ≥40% (77.6 pg/ml, IQR 46.6 to 141.3) than in those with LVEF < 40% (70.0 pg/ml, IQR 47.1 to 100.6; *p* = 0.032), whereas the median sNEP levels did not differ according to current smoking status, all-cause mortality, or MACE (Fig. 1).

### Clinical outcomes

**Fig. 2:**
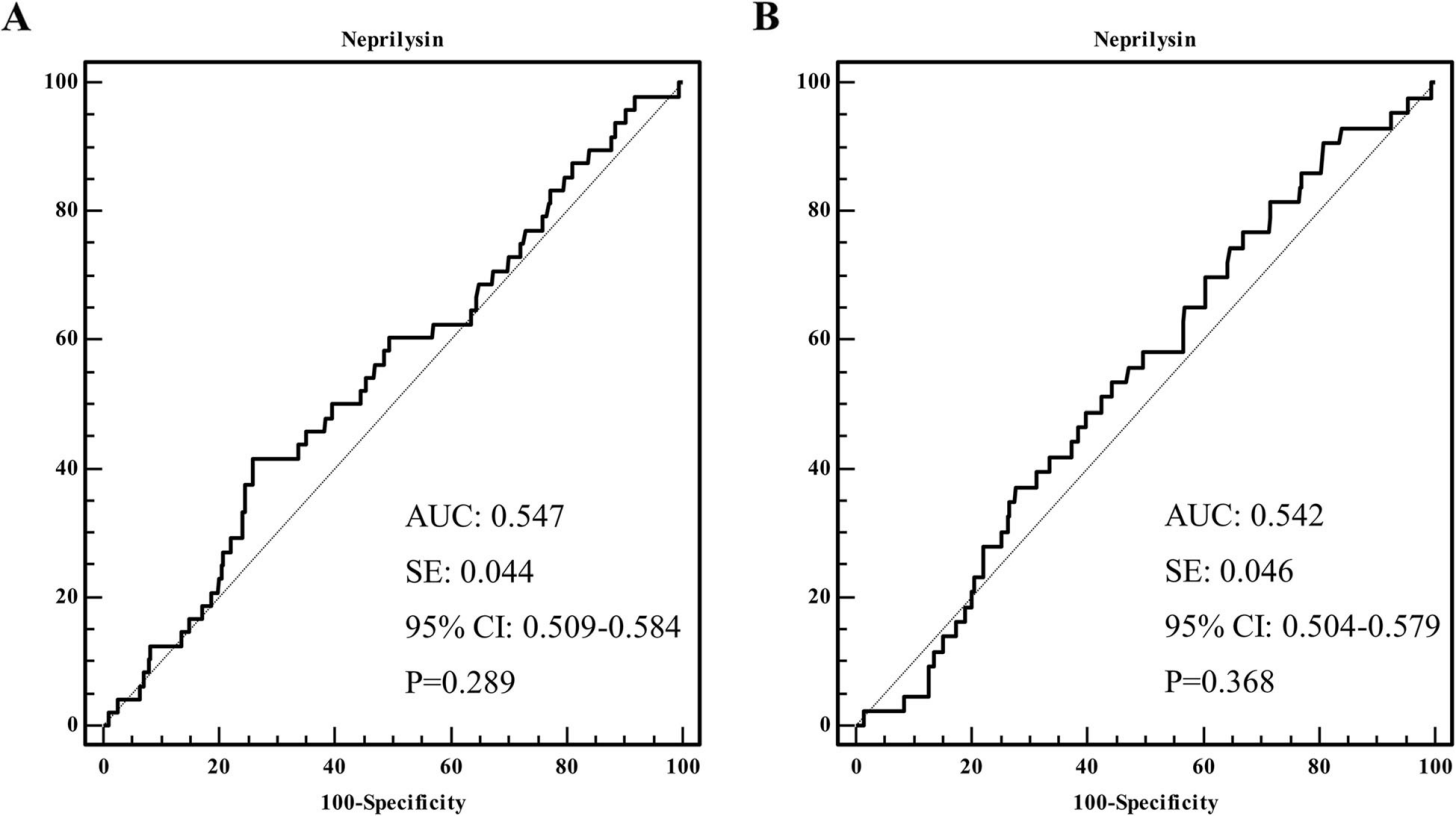
Receiver operating characteristic curve according to the neprilysin level. **a** All-cause death and (**b**) the major cardiovascular events

**Fig. 3:**
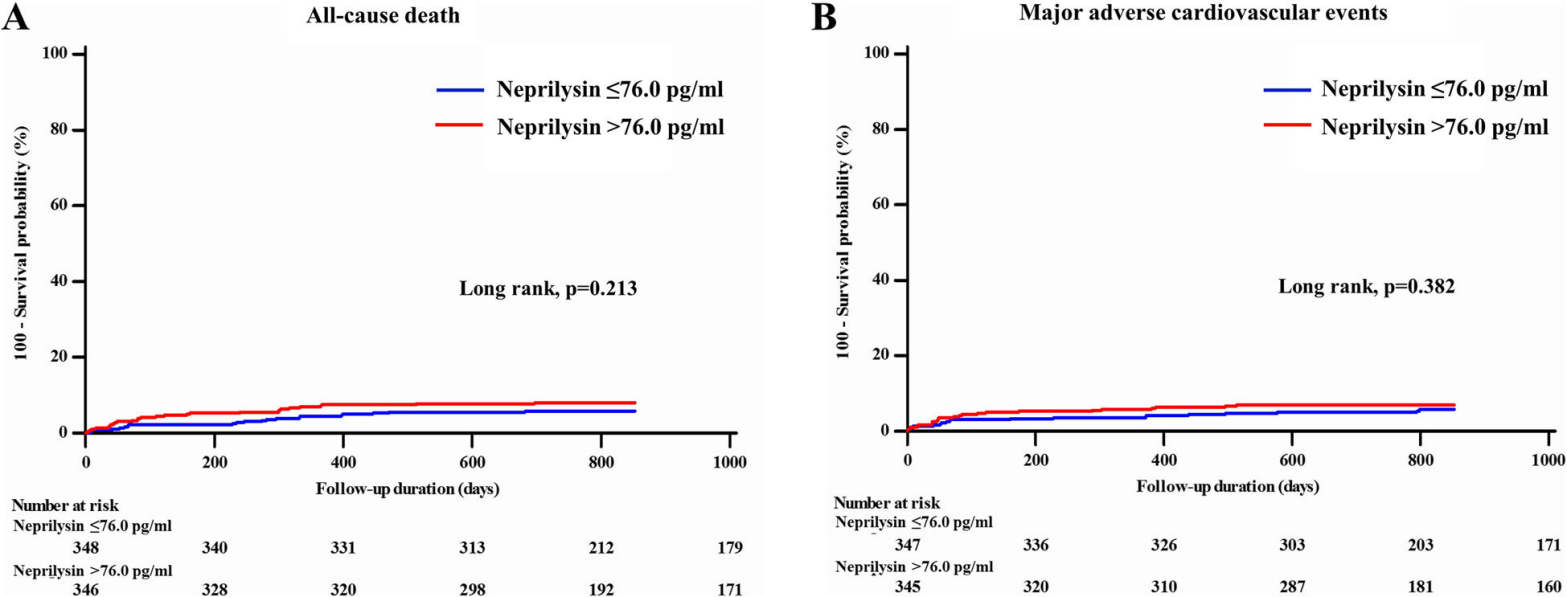
Cumulative incidence rates according to soluble neprilysin level. **a** All-cause death and (**b**) the major cardiovascular events

The median follow-up duration was 28.4 months (IQR 23.4 to 36.2). During the overall follow-up, the incidences of all-cause death and MACE were 6.9 and 6.2%, respectively. The clinical outcomes are presented in Table 2. Among all patients, each clinical outcome and MACE did not differ significantly between the two groups. ROC curve analysis showed that the area under the curve for all-cause death and MACE were 0.547 (95% CI 0.509–0.584, *P* = 0.289) and 0.542 (95% CI 0.504–0.579, *P* = 0.368), respectively (Fig. 2). Figure 3 shows the Kaplan-Meier curves for all-cause death and MACE up to 28.4 months in all patients. When neprilysin was divided into tertiles and quartiles, there were no significant differences in all-cause death or MACE between groups (Table 3). Multivariate Cox regression analysis using various models demonstrated that the higher neprilysin group was not consistently associated with all-cause death or MACE (Table 4). Univariate and multivariate analyses showed that the neprilysin > 76.0 pg/ml group was not a predictor of all-cause death (HR 1.558, 95% CI 0.848–2.863, *P* = 0.153) (Table 5) or MACE (HR 1.450, 95% CI 0.785–2.679, *P* = 0.235).

**Table 2:** Clinical outcomes according to the soluble neprilysin level

Outcome	Neprilysin		Log rank, *P* value
	≤76.0 pg/ml (*n* = 348)	> 76.0 pg/ml (*n* = 346)	
All-cause death	20 (5.7%)	28 (8.1%)	0.213
Cardiovascular death	10 (2.9%)	15 (4.3%)	0.292
Nonfatal myocardial infarction	0 (0%)	1 (0.3%)	0.316
Nonfatal stroke	2 (0.6%)	4 (1.2%)	0.408
Revascularization	5 (1.4%)	5 (1.4%)	0.968
Hospitalization for heart failure	3 (0.9%)	2 (0.6%)	0.664
Major adverse cardiovascular events	19 (5.5%)	24 (6.9%)	0.382

**Table 3:** Clinical outcomes according to the tertile and quartile of the soluble neprilysin level

	Neprilysin				Log rank, *P* value
	< 56.3 pg/ml (*n* = 228)	56.3–111.4 pg/ml (*n* = 235)	> 111.4 pg/ml (*n* = 231)		
All-cause death	15 (6.5%)	13 (5.6%)	20 (8.7%)		0.410
MACE	11 (4.8%)	15 (6.5%)	17 (7.4%)		0.470
	Neprilysin				Log rank, *P* value
	< 46.8 pg/ml (*n* = 173)	46.8–76.0 pg/ml (*n* = 175)	76.0–133.9 pg/ml (*n* = 173)	> 133.9 pg/ml (*n* = 173)	
All-cause death	11 (6.4%)	9 (5.1%)	12 (6.9%)	16 (9.2%)	0.482
MACE	8 (4.6%)	11 (6.3%)	12 (6.9%)	12 (6.9%)	0.760

*MACE* indicates major adverse cardiovascular events

**Table 4:** Association between the neprilysin level with all-cause death and major adverse cardiovascular events

Model	HR (95% CI)	*P* value
All-cause death		
Model 1 (univariate)	1.437 (0.810–2.552)	0.215
Model 2 (age, sex)	1.567 (0.882–2.783)	0.125
Model 3 (age, sex, smoking)	1.575 (0.887–2.798)	0.121
Model 4 (age, sex, LVEF)	1.598 (0.898–2.842)	0.111
Model 5 (age, sex. NT-proBNP)	1.414 (0.789–2.534)	0.244
Model 6^a^	1.701 (0.892–3.247)	0.107
Major adverse cardiovascular events.		
Model 1 (univariate)	1.307 (0.716–2.386)	0.384
Model 2 (age, sex)	1.431 (0.783–2.614)	0.244
Model 3 (age, sex, smoking)	1.431 (0.784–2.613)	0.244
Model 4 (age, sex, LVEF)	1.528 (0.834–2.798)	0.170
Model 5 (age, sex. NT-proBNP)	1.309 (0.705–2.430)	0.394
Model 6^a^	1.624 (0.843–3.127)	0.147

*HR* indicates hazard ratio, *CI* confidence interval, *LVEF* left ventricular ejection fraction, *NT-proBNP* N-terminal pro-B type natriuretic peptide

^a^After adjustment for age, sex, body mass index, hypertension, diabetes mellitus, dyslipidemia, smoking, family history of coronary artery disease, prior stroke, clinical presentation (myocardial infarction vs. angina), LVEF, NT-proBNP, and estimated glomerular filtration rate

**Table 5:** Association between clinical characteristics and the risk of all-cause death during follow-up analyzed by univariate and multivariate Cox proportional hazard model

	Univariate		Multivariate	
	HR (95% CI)	*P* value	HR (95% CI)	*P* value
Age	1.110 (1.075–1.146)	< 0.001	1.077 (1.027–1.130)	0.002
Female	1.748 (0.991–3.085)	0.054	1.133 (0.594–2.160)	0.705
Body mass index	0.917 (0.880–0.956)	< 0.001	1.037 (0.938–1.147)	0.473
Hypertension	1.885 (0.913–3.891)	0.087	1.099 (0.465–2.600)	0.830
Diabetes mellitus	1.414 (0.802–2.495)	0.232		
Dyslipidemia	1.008 (0.547–1.856)	0.980		
Smoking	0.487 (0.228–1.040)	0.063	1.234 (0.523–2.915)	0.631
Family history of CAD	0.527 (0.128–2.171)	0.375		
Chronic kidney disease	2.472 (0.979–6.243)	0.055	0.957 (0.303–3.018)	0.940
Prior stroke	1.436 (0.644–3.200)	0.377		
Acute myocardial infarction	4.320 (2.153–8.671)	< 0.001	3.191 (1.502–6.776)	0.003
Left ventricular ejection fraction	0.947 (0.929–0.965)	< 0.001	0.967 (0.942–0.994)	0.015
eGFR	0.960 (0.948–0.973)	< 0.001	0.983 (0.960–1.007)	0.173
NT-proBNP	1.000 (1.000–1.000)	< 0.001	1.000 (1.000–1.000)	0.420
Multivessel disease	1.009 (0.548–1.857)	0.978		
Number of total stents	0.992 (0.764–1.290)	0.954		
Mean diameter of stents	0.745 (0.363–1.528)	0.421		
Total length of stents	1.006 (0.998–1.013)	0.168		
Neprilysin > 76.0 pg/ml	1.437 (0.810–2.552)	0.215	1.558 (0.848–2.863)	0.153

*HR* indicates hazard ratio, *CI* confidence interval, *CAD* coronary artery disease, *eGFR* estimated glomerular filtration rate, *NT-proBNP* N-terminal pro-B-type natriuretic peptide

### Subgroup analysis

**Fig. 4:**
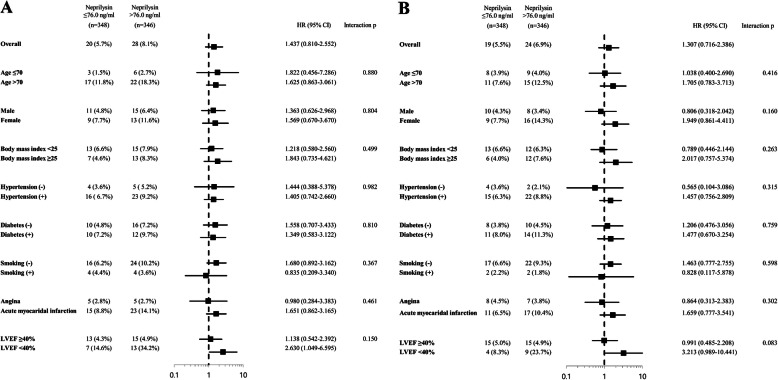
Comparison of outcomes between soluble neprilysin level higher and lower group according to subgroups. **a** All-cause death and (**b**) the major cardiovascular events

**Fig. 5:**
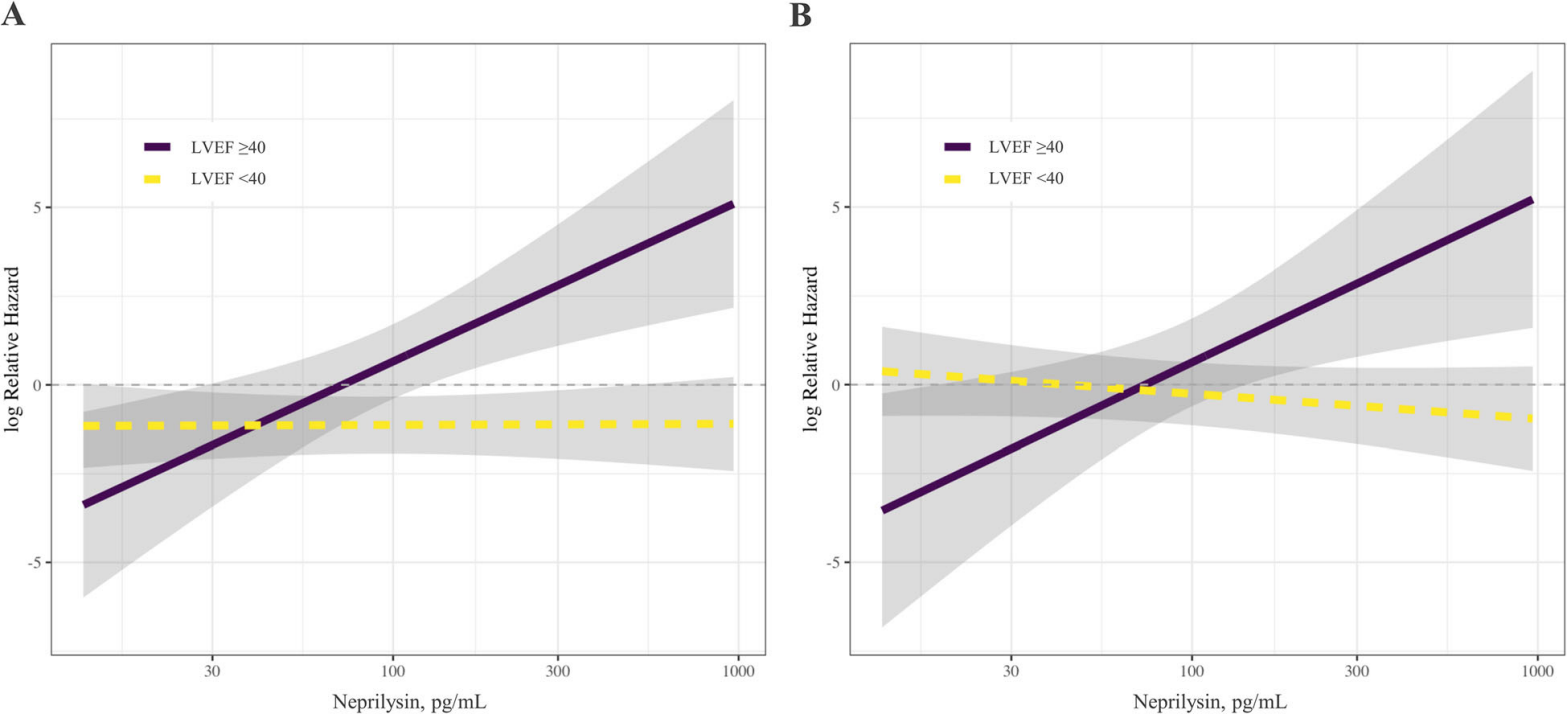
Longitudinal associations of soluble neprilysin and risk of outcomes according to LVEF after multivariable adjustment. **a** All-cause death and (**b**) the major adverse cardiovascular events. Solid purple lines represent the calculated log relative hazard in patients with left ventricular ejection fraction less than 40, and the dashed yellow line represents the log relative hazard for patients with left ventricular ejection fraction equal to or greater than 40. Shaded gray areas represent 95% confidence intervals. LVEF = left ventricular ejection fraction

We stratified the overall patients by age, sex, and important comorbidities. This subgroup analysis revealed consistent trends irrespective of each subgroup except the LVEF < 40% group (Fig. 4). The LVEF < 40% group had a trend of a higher rate of all-cause death in the neprilysin > 76.0 pg/ml group without a statistically significant interaction (HR 2.630, 95% CI 1.049–6.595, *P* for interaction = 0.150) and a marginal trend of a higher rate of MACE (HR 3.213, 95% CI 0.989–10.441, *P* for interaction = 0.083). In the analysis for an association between sNEP levels in continuous scales and relative hazard for outcomes, the Cox proportional hazard model results suggest that sNEP level is not a significant factor by itself when considered without its interaction with LVEF (*P* = 0.094). However, the detailed analysis indicates that increased sNEP levels are associated with a higher risk of all-cause death and cardiovascular adverse events for patients with decreased LVEF but not for those with preserved LVEF (*P* for interaction = 0.003 for all-cause mortality, *P* for interaction = 0.004 for MACE) (Fig. 5).

## Discussion

The current study found that serum sNEP levels are not associated with mortality or cardiovascular outcomes during follow-up. However, increased sNEP levels tend to be associated with a higher risk of all-cause mortality or cardiovascular events in patients with reduced LVEF but not in patients with preserved LVEF.

There are 3 types of natriuretic peptides, atrial, brain or B-type, and C-type [19–22]. Natriuretic peptides have potent effects on sodium and fluid balance, and they have a very important role in various heart diseases [1, 23, 24]. Circulating natriuretic peptides are cleared through 2 essential mechanisms: one is natriuretic peptide receptor-mediated clearance, and the other is degradation by enzymes. NEP is one of the most important proteases that cleaves human natriuretic peptides, and the cleavage sites on natriuretic peptides have been well established [1, 25]. NEP was isolated for the first time in the 1970s as an endopeptidase in renal proximal tubule cells of rabbits [26, 27]. Since then, several brilliant studies have established the function of NEP. NEP is not only a potent hydrolyzer of natriuretic peptides but also a membrane-bound enzyme that degrades a large number of vasodilator peptides, including adrenomedullin, bradykinin, angiotensin, substance P, endothelin-1, and amyloid-beta protein [1, 28–31]. Although NEP is a membrane-bound metalloprotease, it can be released from the cell membrane. The production of sNEP is known to occur as a consequence of proteolytic cleavage of the extracellular domain or exosomal release dependent on A Disintegrin And Metalloprotease-17 (ADAM-17) [1, 10, 32]. In addition, NEP may exist as either a transmembrane or soluble form, and sNEP retains its catalytic activity [1, 10, 33].

Circulating sNEP has been reported as a biomarker surrogate in patients with HF. In chronic ambulatory HF patients with at least 1 hospitalization for HF or reduced LVEF, sNEP was positively associated with cardiovascular mortality and morbidity [10]. In comprehensive multivariable analyses, sNEP remained to be significantly associated with both composite endpoint and cardiovascular mortality independent of conventional clinical risk factors and NT-proBNP. Elevated sNEP levels predicted an increased risk of recurrent admission for HF in the same study population with chronic HF [34]. In addition, the prognostic value of sNEP has been evaluated in acute decompensated HF [11]. Admission sNEP concentration was associated with short- and long-term outcomes in acute HF. On the other hand, another study could not confirm an association between sNEP levels and cardiovascular mortality or hospitalization for HF in patients with HFpEF [14]. In addition, sNEP levels showed no significant relationship with myocardial infarct size and 1-year adverse outcomes in patients with ST-elevation myocardial infarction [13]. More recently, in a large community-based cohort, the investigators reported that sNEP did not correlate with natriuretic peptide levels and was not independently associated with adverse outcomes [35].

The present study aimed to demonstrate the prognostic value of sNEP levels in patients with CAD undergoing PCI. To the best of our knowledge, this study is the first analysis of sNEP as a prognostic biomarker in all-comer PCI patients. In particular, most of the patients were relatively high-risk patients with acute coronary syndrome. Consequently, the results of this study showed no association between sNEP levels and long-term mortality or major cardiovascular events. However, in the subgroup analysis, CAD patients with reduced LVEF seemed to show a positive association between circulating sNEP levels and mortality and MACE, while there was no association between circulating sNEP levels and preserved LVEF. These findings are consistent with the results of previous studies regarding sNEP as a biomarker for long-term clinical outcomes in patients with HFrEF and HFpEF. However, this finding may not be conclusive because only 12.4% of patients who were included in our study presented with LVEF less than 40%, and the subgroup analysis did not have strong statistical power.

Nevertheless, the present results may be translated meaningfully by expanding further studies in the future. To summarize, in previous studies, sNEP levels were associated with long-term prognosis in patients with HFrEF or acute HF, whereas they were not associated with outcomes in patients with HFpEF. In addition, the combination of a NEP inhibitor with angiotensin receptor blocker demonstrated superior clinical outcomes in patients with HFrEF and a greater reduction in the NT-proBNP concentration in patients with acute decompensated HF than did an angiotensin-converting enzyme inhibitor alone, while the combination therapy did not result in a significantly lower rate of the clinical outcome among patients with HFpEF. On the basis of these previous results and our results, serum sNEP levels can be used to predict the long-term prognosis of patients with ischemic heart disease accompanied by chronic HFrEF or acute HF or CAD patients with reduced LVEF, and further findings will support the importance of NEP inhibition in those populations concerning better clinical outcomes.

Previous studies showed that sNEP levels were lower in HFpEF compared with controls without HFpEF, and the lowest sNEP tertile group had the highest prevalence of diastolic dysfunction in general population [6, 35]. The other study found that plasma NEP concentration was lower in non-acute decompensated HF than acute decompensated HF and higher in chronic HF than acute decompensated HF [36]. The same study demonstrated different patterns in distributions of immunoreactive N-type natriuretic peptides or circulating neprilysin activity. The action mechanisms of NEP, such as interaction between NEP and numerous vasoactive peptides or relationship between sNEP activity and concentration, have remained unknown. Our study found that serum sNEP levels were lower in CAD patients with reduced LVEF compared with those with preserved LVEF. Further investigations are needed to elucidate this paradoxical finding of low concentration and potential prognostic values in patients with reduced LVEF. Furthermore, patients with higher neprilysin level group were younger than lower group. Similar to this study, there was a study that age was negatively associated with level of neprilysin [34]. The mechanism of the negative correlation between age and neprilysin have remained unknown, and further evaluation is needed.

The present study has some limitations. First, we could not assess the biological activity of serum NEP. Therefore, we could not analyze the relationship between serum levels and biological activity and could not adjust these relationships. Second, we collected a blood sample only once per patient at the time of coronary angiography and could not evaluate the changes in sNEP levels. We cannot exclude that changes in sNEP levels are related to long-term clinical outcomes in ischemic heart disease. Third, the blood samples were stored for 1 ~ 2 years prior to analysis because the ELISAs were performed once after all patients were enrolled. Therefore, some unexpected alterations might have occurred, and these changes could affect the results. Finally, the present study included patients with CAD undergoing PCI, and the results of our study may not be directly applicable to patients with other categories of the disease entity.

## Conclusions

Serum sNEP levels are not associated with the risk of all-cause death and cardiovascular events in patients with CAD undergoing PCI. Among patients with LVEF less than 40%, increased sNEP levels seem to be associated with a higher risk of all-cause death. The prognostic value of sNEP levels may be different depending on whether LVEF is preserved or reduced in ischemic heart disease. Further large-scale studies are needed to verify the results of the present study, and large clinical trials will translate these results into clinical practice to determine whether a NEP inhibitor improves the clinical outcomes in ischemic heart disease with or without decreased left ventricular systolic function.

## Data Availability

The datasets used and/or analyzed during the current study are available from the corresponding author on reasonable request.

## Abbreviations

CAD: Coronary artery disease;
CI: Confidence interval
ELISA: Enzyme-linked immunosorbent assay
HF: Heart failure
HFpEF: Heart failure and preserved ejection fraction
HFrEF: Heart failure and reduced ejection fraction
HR: Hazard ratio
IQR: Interquartile range
LVEF: Left ventricular ejection fraction
MACE: Major adverse cardiovascular events
NEP: Neprilysin
PCI: Undergoing percutaneous coronary intervention
ROC: Receiver operating characteristic
sNEP: Soluble neprilysin
